# Femoral Head Coverage Assessment in Healthy Children Younger than 6 Years

**DOI:** 10.1155/2022/6058746

**Published:** 2022-07-22

**Authors:** Suvorov Vasyl, Filipchuk Viktor, Zyablovskyi Evhen

**Affiliations:** ^1^Department of Joint Diseases in Children and Adolescents, SI, The Institute of Traumatology and Orthopedics By Nams of Ukraine, Kyiv, Ukraine; ^2^The Center of Radiology, Department of Radiation and Radionuclide Diagnostics, National Specialised Hospital “OHMATDYT”, Kyiv, Ukraine

## Abstract

**Introduction:**

Developmental dysplasia of the hip (DDH) is one of the commonest hip joint pathologies in children; to treat it properly, hip surgeons should know the normal femoral head (FH) coverage by the acetabulum. This paper aims to assess the femoral head coverage in healthy children younger than 6 years.

**Methods:**

270 hip joint CT scans were selected, and digital pelvic models were created according to these scans. FH coverage by the five acetabular regions was assessed according to patient's age and sex.

**Results:**

Normal reference values of FH coverage by different acetabular regions were obtained. It was found that the growth process of different acetabular regions occurs nonlinearly with the periods of acceleration. Anterior and superior-anterior acetabular regions grow more intensively in boys up to 3 years old and between 4 and 5 years old both in boys and girls; superior-posterior, posterior-superior, and posterior-inferior acetabular regions grow more intensively in boys and girls up to 3 years old and between 4 and 5 years old (*p* ≤ 0.005). The following sex differences in FH coverage by the acetabulum were found: more superior-anterior FH coverage was found in boys and posterior FH coverage in girls (*p* ≤ 0.005).

## 1. Introduction

Developmental dysplasia of the hip (DDH) is one of the commonest hip joint pathologies in pediatric orthopedist practice [1]; without treatment, it leads to early hip arthritis [2]. In younger patients, treatment is usually nonsurgical, but in older patients (who began to walk), surgical treatment is preferable [3]. Among all surgical options, the best results were obtained after pelvic osteotomies [4–6]. In patients under 6 years of age (when the most active acetabular development occurs [7, 8]), three pelvic osteotomies are widely used—Salter, Pemberton, and Dega osteotomies. These osteotomies may improve both anterior-superior or posterior-superior femoral head (FH) coverage [9, 10]. To apply these pelvic osteotomies more effectively, it is necessary to understand the normal acetabulum morphology and its maturation process in healthy children of 1–6 years old.

Previously, acetabulum morphology was evaluated by other authors, but in adults [11–22], the youngest age in these studies was 8 years. [16]. There are no studies dedicated to evaluating acetabulum morphology in children under 6 years. As we know this study is the first one, the reliable way to assess acetabular morphology is to assess the FH coverage by the acetabulum as described by others [12, 13, 16].

The goal of this study is to assess the femoral head coverage by the acetabulum in healthy children younger aged 1–6 years.

## 2. Methods

Pelvic CT scans of children aged 1–6  years without hip joint pathologies were selected for this study. CT scans were selected from the “OHMATDYT” hospital database for the period of 2010–2020. These patients did not have any hip joint pathologies; all of them underwent abdominal and pelvic CT scanning to detect neoplasms metastases. These pelvic CT scans with a slice thickness <1.5 mm (for better visualization of pelvic bones) were selected for further investigation. The scanning was performed on “Siemens SOMATOM Definition AS, USA.”

Totally, 270 CT scans of 135 patients were selected for further work: 156 hip joint CT scans of 78 male patients and 114 hip joint CT scans of 57 female patients.

Selected CT scans were transported into Mimics 20.0 software (Materialize Inc., Leuven, Belgium), and automatic segmentation of pelvic bones was performed. Digital pelvic models were exported from Mimics software in STL format. Furthermore, these models were transported into custom-made software for the FH coverage assessment.

**Figure 1:**
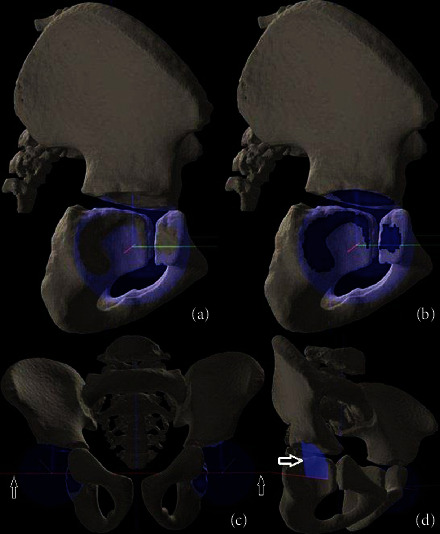
The process of FH coverage assessment by the acetabulum. (a) the virtual sphere; (b) the contact spot between the sphere and acetabular edge; (c) the reference line (arrows); (d) an intersection angle (arrow).

FH coverage was assessed by fitting the virtual sphere into the acetabulum with the least-squares method. Then, the contact between the virtual sphere and acetabular borders was highlighted (with a contact spot). After that, the reference line was drawn through the centers of both spheres. Finally, intersection angles from the sphere's center between the reference line and acetabular edges were measured circumferentially. The measurement step was 1°, so each virtual sphere had 360 contact points with the acetabular edge (both with pelvic bones and triradiate cartilage limbs). Thus, 360 angles were measured. This data was represented both as numerical values and graphically displayed in the chart. The numerical values were exported to Microsoft Corporation (2007) and Microsoft Excel for further analysis. The pelvic spatial alignment (as described by others [12, 13]) was not performed. This was not necessary because pelvic bones in children are separated with triradial cartilage limbs that allow identifying each pelvic bone in the acetabulum without any reference points. The process of FH coverage assessment is shown in Figure 1.

**Figure 2:**
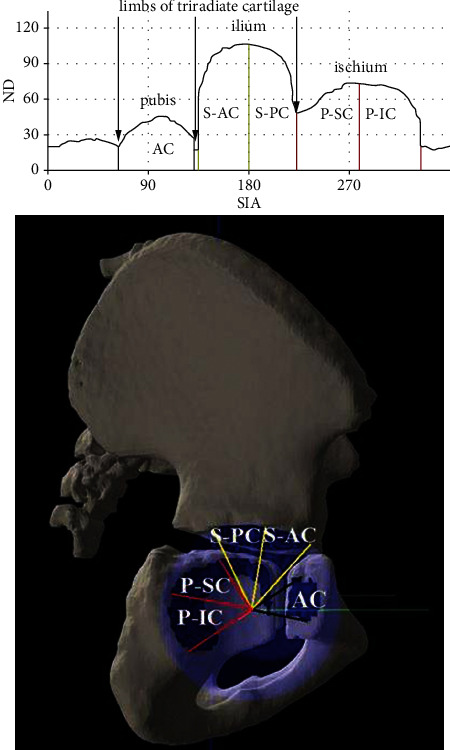
The graphical presentation of FH coverage by different acetabular regions. AC: anterior coverage; S-AC: superior-anterior coverage; S-PC: superior-posterior coverage; P-SC: posterior-superior coverage; P-IC: posterior-inferior coverage.

The FH coverage was assessed by the pubis, ilium, and ischium bones (anterior, superior, and posterior acetabular coverage, respectively). For a more detailed assessment, the regions of superior and posterior acetabulum coverage were further divided into the superior-anterior, superior-posterior, posterior-superior, and posterior-inferior regions. Thus, FH coverage by the 5 acetabular regions (anterior, superior-anterior, superior-posterior, posterior-superior, and posterior-inferior) was evaluated. FH coverage by different acetabular regions is presented in Figure 2.

Before the assessment of the FH coverage in selected children, we had the following learning curve. The FH coverage in other 70 children (140 hip joints) of both genders 1–6 years old without hip joint pathologies was assessed. This practice taught us how to overcome with more shallow acetabulum in younger children while fitting in it the virtual sphere. Also, after this training, we have decided to select only CT scans with slice thickness <1.5 mm for better pelvic bone visualization for further FH coverage assessment.

### 2.1. Statistical Analysis

Data were normalized as follows: 360 numerical values (intersection angles values) were entered for each child in Microsoft Corporation (2007), Microsoft Excel. Then, all children and the abovementioned numerical values were sorted according to their sex (male and female children separately) and age (from 1 to 6 years old). Furthermore, these numerical values were divided according to the FH coverage by an anterior acetabular region (covered by pubis bone), superior region (ilium bone), and posterior region (ischium bone). The numerical values of superior and posterior FH coverage were divided into two parts, which reflected the superior-anterior, superior-posterior, posterior-superior, and posterior-inferior FH coverage. The arithmetic means in each of the five FH coverage regions were calculated according to the child's sex and age. These arithmetic mean values presented themselves as the raw data for further statistical analysis.

To determine normal FH coverage reference values, arithmetic mean values of the FH coverage by different acetabular regions were normally distributed according to children's age and sex. The mean, maximal, minimum values and standard deviation were calculated for each group.

To determine the development intensity in acetabular regions, the mean values of the FH coverage by the same acetabular region in children of the adjacent age and the same sex were compared. The null hypothesis (Ho) was the absence of intensive development of certain acetabular regions in a certain age period. An alternative hypothesis (Ha) was the presence of such intensive development. The hypothesis was tested with Student's *t*-test. The level of significance (*α*) was set at 5%. At a “*p*” value <0.05, the development of a certain acetabular region in a certain age period was considered intensive.

Sex differences in the FH coverage by the same acetabular region in children of the same age were assessed similarly. The null hypothesis (Ho) was the absence of sex difference in the FH coverage by the same acetabular region in male and female children of the same age. An alternative hypothesis (Ha) was the presence of such a difference. The hypothesis was tested with Student's *t*-test. The level of significance (*α*) was set at 5%. At a “*p*” value <0.05, the sex difference in the FH coverage by the same acetabular region in male and female children of the same age was considered significant.

To assess the reproducibility of our measurements the intrarater correlation was calculated by the same rater (Suvorov Vasyl). FH coverage by different acetabular regions was assessed according to the method described above (in the “Methods” section). 4 months after the initial assessment, an intrarater correlation was calculated using intraclass correlation (ICC). The reproducibility was considered poor with an ICC value <0.50; moderate-with 0.50 ≤ ICC < 0.75; good-with 0.75 ≤ ICC < 0.90; and excellent-with ICC ≥0.90.

Initial data collection and processing were performed in Microsoft Corporation. (2007), Microsoft Excel. Statistics calculations were performed by JASP Team (2020), JASP (version 0.11.1.0) (Computer software). Results with a *p* value ≤0.05 were considered statistically significant.

## 3. Results

Normal reference values of FH coverage by different acetabular regions in children according to their age and sex are shown in Table 1.

According to the growth of different acetabular regions it was revealed that the anterior region grows more intensively in male children between 1-2 and 4-5 years (*p* ≤ 0.05); in female children, it grows rapidly between 1 and 2 years, then there is retardation up to 3 years of age (*p* ≤ 0.05). The superior-anterior acetabular region grows intensively in male children between 2-3 and 4-5 years and in female children between 4 and 5 years (*p* ≤ 0.05). The superior-posterior acetabular region grows intensively in male children up to 3 y.o. and between 4 and 5 years, in female children up to 2 y.o. and between 4 and 5 years (*p* ≤ 0.05). The posterior-superior acetabular region grows intensively in male and female children up to 3 y.o. and between 4 and 5 years (*p* ≤ 0.05). The posterior-inferior acetabular region grows intensively in male and female children for up to 3 y.o. (*p* ≤ 0.05). The comparison of FH coverage by different acetabular regions in male and female children according to their age is shown in Table 2.

Regarding the sex differences in FH coverage in male and female children, it was found that superior-anterior FH coverage is more in male children at 3 y.o. (*p* ≤ 0.05). Contrarily, it was revealed that the posterior-superior FH coverage is more in girls at 2 and 6 y.o. (*p* ≤ 0.05); the posterior-inferior FH coverage is more in girls at 2, 4, and 5 y.o. (*p* ≤ 0.05). Sex differences in FH coverage in children according to their age are shown in Table 3.

According to the reproducibility of our measurements, the moderate-to-good intrarater agreement was seen in all cases. The table of intraclass correlation according to the patient's age and FH coverage region is presented in Table 4.

## 4. Discussion

This study aimed to investigate the femoral head coverage by the five acetabular regions in healthy children of 1–6 years old. In this study, FH coverage was assessed from the standpoint of pelvic osteotomies application for DDH treatment, as this pathology is the commonest hip joint pathology in children 1–6 years old, which requires pelvic osteotomies. It is important for the hip surgeon to know the normal reference values of the FH coverage, as this allows for differentiating pathological conditions from the normal extremes and planning hip joint reconstructive surgeries. However, if needed, the results of this study are also applicable for other pathologies that involve hip joint in children of 1–6 years old (cerebral palsy, multiple epiphyseal dysplasia, and secondary acetabular dysplasia in Legg–Calve–Perthes disease).

The method of FH coverage assessment, described in this article, allows hip surgeons to appreciate acetabular morphology indirectly and to compare it with reference values. These reference values will be useful when determining the type of acetabular deficiency and for better preoperative planning; however, pelvic CT scans are needed for this.

Analyzing the acetabulum maturation process, we have found out that the most intensive acetabular growth occurs during the first 5 years of life, which is consistent with the results of Li, L. Y., and Novais [7, 8, 17]. This justifies the early application of reconstructive pelvic osteotomies for DDH treatment. Also, this may explain better results after pelvic osteotomies in younger patients compared to older ones [23–28].

Regarding sex differences in the FH coverage, we have revealed that posterior FH coverage is more in female children and anterior FH coverage is more in male children. Our findings are in concordance with the results of other authors about gender differences in hip joint morphology in adults [12, 13, 16]. This may raise the question if these differences arise from childhood.

The drawbacks of this work are as follows: (1) only the boney part of the acetabulum was included in this study as CT scans were evaluated; this does not allow for fully appreciating FH coverage in children due to a large amount of chondral tissue in the acetabulum (that is not seen on CT scans). Thus, MRI studies are preferable to CT scans for the assessment of FH coverage in children. However, CT scans instead of MRI are widely used in children for different abdominal and pelvic cavities pathologies, so they were chosen as research objects in this study. (2) The method of FH coverage assessment described in this article is sophisticated. It may be inconvenient for routine application as pelvic CT scans and reference data of the FH coverage by different acetabular regions are needed for it. Moreover, the results and reference values described in this article may be different in other countries (because of possible differences in pelvic morphology). (3) The validation of our technique was not performed.

## 5. Conclusion

After the assessment of the FH coverage by different acetabular regions, normal reference values of this coverage were obtained. These reference values may be used by hip surgeons during preoperative planning of pelvic osteotomies for DDH treatment (or for other hip joint pathologies) in patients of 1–6 years old. Evaluating the maturation process of different acetabular regions, it was found that it occurs nonlinearly with periods of acceleration (up to 3 y.o. and between 4 and 5 y.o.); this also should be taken into account during preoperative planning (to prevent overcorrection in those regions that still growth). Assessing sex differences in the FH coverage, more intensive superior-anterior coverage in male children and posterior coverage in female ones were found. This information should be considered by the hip surgeon during routine preoperative planning without CT scans (to prevent overcorrecting FH coverage in those regions that better grow).

## Figures and Tables

**Table 1:** Reference values of normal FH coverage by different acetabular regions in boys and girls aged 1–6 y.o.

FHC	Male		Mean (±std. dev.) Minimum/maximum			Female		Mean		
								(±std. dev.)		
								Minimum/maximum		
y.o.	AC	S-AC	S-PC	P-SC	P-IC	AC	S-AC	S-PC	P-SC	P-IC
1	19.44 (±15.7) 0/38	89.05 (±3.76) 81/100	86.29 (±3.38) 77/94	50.14 (±5.12) 44/62	50.2 (±5.06) 39/60	14.54 (±17.6) 0/41	88.9 (±5.0) 79/97	84.72 (±5.43) 73/92	49.04 (±6.79) 34/58	51.31 (±5.99) 42/64
2	28.57 (±9.98) 5/41	92.35 (±6.74) 83/103	90.78 (±5.28) 84/99	53.92 (±3.56) 47/60	53.42 (±5.41) 43/60	33.93 (±4.5) 25/41	100.87 (±30.09) 78/211	91.06 (±6.14) 76/98	56.93 (±5.3) 50/66	57.87 (±5.94) 50/67
3	36.1 (±4.48) 27/43	98.2 (±4.2) 90/107	96.45 (±3.45) 89/103	63.1 (±2.8) 57/68	60.85 (±4.03) 53/68	30.72 (±15.28) 0/47	94.22 (±5.48) 84/104	92.5 (±7) 70/100	61.16 (±6.47) 49/72	63.22 (±6.3) 52/71
4	35.07 (±8.97) 11/44	96.15 (±4.28) 89/105	95.57 (±3.94) 88/103	61.92 (±3.59) 57/71	62.03 (±3.9) 55/68	34.54 (±11.28) 8/48	95.68 (±4.86) 87/106	94.63 (±5.42) 83/102	61.45 (±7.31) 42/71	63.45 (±7.23) 47/71
5	41.61 (±4.56) 35/50	100.15 (±5.37) 90/109	99.53 (±3.92) 91/106	66.19 (±3.8) 58/73	64.11 (±4.06) 59/75	41.24 (±9.83) 28/57	99.35 (±4.41) 92/104	100.85 (±3.69) 94/106	69.64 (±7.84) 59/85	66.5 (±5.53) 59/78
6	41.72 (±4.8) 30/51	100.63 (±4.12) 92/107	100.44 (±4.39) 92/110	68.86 (±5.57) 55/79	66.77 (±5.13) 56/76	43.18 (±6.93) 30/54	99.54 (±4.87) 91/108	101.04 (±4.69) 92/110	71.77 (±5.25) 61/84	69.9 (±3.57) 63/79

FHC: femoral head coverage; y.o.: years old; AC: anterior coverage; S-AC: superior-anterior coverage; S-PC: superior-posterior coverage; P-SC: posterior-superior coverage; P-IC: posterior-inferior coverage; std. dev: standard deviation.

**Table 2:** The comparison of FH coverage by different acetabular regions in children according to age and sex.

Male							Female						
Anterior coverage y.o							Anterior coverage						
y.o.	1	2	3	4	5	6	y.o.	1	2	3	4	5	6
1	—	0.019	<0.001	<0.001	<0.001	<0.001	1	—	<0.001	<0.001	<0.001	<0.001	<0.001
2	—	—	0.053	0.234	0.004	<0.001	2	—	—	0.0414	0.987	0.032	<0.001
3	—	—	—	0.302	0.002	0.002	3	—	—	—	0.074	0.04	0.011
4	—	—	—	—	<0.001	0.003	4	—	—	—	—	0.063	0.005
5	—	—	—	—	—	0.683	5	—	—	—	—	—	0.724
6	—	—	—	—	—	—	6	—	—	—	—	—	—

Superior-anterior coverage							Superior-anterior coverage						
y.o.	1	2	3	4	5	6	y.o.	1	2	3	4	5	6
1	—	0.12	<0.001	<0.001	<0.001	<0.001	1	—	0.152	0.012	<0.001	<0.001	<0.001
2	—	—	0.013	0.057	0.004	<0.001	2	—	—	0.475	0.452	0.845	0.813
3	—	—	—	0.193	0.267	0.206	3	—	—	—	0.806	0.099	0.021
4	—	—	—	—	0.02	<0.001	4	—	—	—	—	0.018	0.007
5	—	—	—	—	—	0.878	5	—	—	—	—	—-	0.365
6	—	—	—	—	—	—	6	—	—	—	—	—	—

Superior-posterior coverage							Superior-posterior coverage						
y.o.	1	2	3	4	5	6	y.o.	1	2	3	4	5	6
1	—	0.027	<0.001	<0.001	<0.001	<0.001	1	-	0.028	0.004	<0.001	<0.001	<0.001
2	—	—	0.008	0.042	<0.001	0.002	2	—	—	0.518	0.175	<0.001	<0.001
3	—	—	—	0.156	0.036	0.011	3	—	—	—	0.397	0.002	0.004
4	—	—	—	—	<0.001	0.003	4	—	—	—	—	0.004	<0.001
5	—	—	—	—	—	0.955	5	—	—	—	—	—	0.341
6	—	—	—	—	—	—	6	—	—	—	—	—	—

Posterior-superior coverage							Posterior-superior coverage						
y.o.	1	2	3	4	5	6	y.o.	1	2	3	4	5	6
1	—	<0.001	<0.001	<0.001	<0.001	<0.001	1	—	0.016	<0.001	<0.001	<0.001	<0.001
2	—	—	<0.001	<0.001	<0.001	<0.001	2	—	—	0.013	0.004	<0.001	<0.001
3	—	—	—	0.45	0.068	0.003	3	—	—	—	0.901	0.018	<0.001
4	—	—	—	—	<0.001	<0.001	4	—	—	—	—	0.003	<0.001
5	—	—	—	—	—	0.3	5	—	—	—	—	—	0.784
6	—	—	—	—	—	—	6	—	—	—	—	—	—

Posterior-inferior coverage							Posterior-inferior coverage						
y.o.	1	2	3	4	5	6	y.o.	1	2	3	4	5	6
1	—	0.015	<0.001	<0.001	<0.001	<0.001	1	—	0.049	<0.001	<0.001	<0.001	<0.001
2	—	—	0.005	<0.001	<0.001	<0.001	2	—	—	0.013	0.03	0.005	<0.001
3	—	—	—	0.273	0.068	0.003	3	—	—	—	1	0.516	0.002
4	—	—	—	—	0.08	0.005	4	—	—	—	—	0.296	<0.001
5	—	—	—	—	—	0.249	5	—	—	—	—	—	0.051
6	—	—	—	—	—	—	6	—	—	—	—	—	—

y.o.: years old.

**Table 3:** Sex differences in FH coverage in children according to their age.

Male/female (*p* value)						
Coverage y.o.	1 y.o.	2 y.o.	3 y.o.	4 y.o.	5 y.o.	6 y.o.
Anterior	0.835	0.163	0.269	0.94	0.958	0.248
Superior-anterior	0.98	0.293	0.048 (M)	0.599	0.601	0.71
Superior-posterior	0.463	0.671	0.092	0.789	0.328	0.174
Posterior-superior	0.982	0.037 (F)	0.277	0.649	0.076	0.014 (F)
Posterior-inferior	0.258	0.019 (F)	0.109	0.456	0.037 (F)	0.01 (F)

y.o.: years old.

**Table 4:** Intraclass correlation values of normal FH coverage by different acetabular regions in male and female children under 6 y.o.

ICC	Male children				
Age (y.o) Coverage	AC	S-AC	S-PC	P-SC	P-IC
1	0.83	0.65	0.69	0.73	0.74
2	0.86	0.80	0.86	0.64	0.81
3	0.79	0.79	0.72	0.68	0.75
4	0.86	0.71	0.76	0.67	0.72
5	0.79	0.79	0.68	0.71	0.73
6	0.78	0.76	0.69	0.73	0.8

ICC	Female children				
					
1	0.86	0.69	0.77	0.82	0.83
2	0.76	0.83	0.83	0.87	0.86
3	0.86	0.84	0.86	0.90	0.76
4	0.87	0.76	0.85	0.84	0.72
5	0.84	0.77	0.78	0.84	0.85
6	0.89	0.76	0.74	0.82	0.71

ICC: intraclass correlation; y.o.: years old; AC: anterior coverage; S-AC: superior-anterior coverage; S-PC: superior-posterior coverage; P-SC: posterior-superior coverage; P-IC: posterior-inferior coverage.

## Data Availability

Pelvic CT scans, digital pelvic models, and software for femoral head coverage used to support the findings of this study are available from the corresponding author upon request. Other data used to support the findings of this study are included within the article.
